# Comparison of survivors and nonsurvivors in 27 confirmed injectional anthrax cases from the 2009 outbreak in Scotland

**DOI:** 10.1186/cc12010

**Published:** 2013-03-19

**Authors:** M Booth, L Donaldson, C Xizhong, S Junfeng, P Eichacker

**Affiliations:** 1 Glasgow Royal Infirmary, Glasgow, UK; 2 National Institutes for Health, Bethesda, MD, USA

## Introduction

From December 2009 to December 2010, 47 patients in Scotland presented with confirmed anthrax infection manifested by soft tissue disease related to heroin injection. These cases represent the first known outbreak of a recently recognized form of anthrax, termed injectional anthrax, which appears to be associated with a high mortality rate (28% in confirmed cases from the UK outbreak). While epidemiologic data from this outbreak have been published, no report has systematically described findings in patients at presentation or compared these findings in nonsurvivors and survivors.

## Methods

To better describe injectional anthrax, we developed a questionnaire and sent it to clinicians who had cared for confirmed cases during the outbreak. Completed questionnaires describing 27 patients, 11 nonsurvivors and 16 survivors, were returned.

## Results

In preliminary analysis of categorical data, a significantly (Fisher exact test) greater proportion of patients with compared with without the following findings did not survive; history of alcohol use (*P *= 0.05); the presence of lethargy (*P *= 0.01), confusion (*P *= 0.03), nausea (*P *= 0.04), abdominal pain (*P *= 0.02), or the need for vasopressors (*P *= 0.002), oxygen, mechanical ventilation, or steroids (all *P *= 0.004) at presentation; and excessive bleeding at surgery (*P *= 0.01). Initial analysis of continuous data demonstrated that, compared with survivors at presentation, nonsurvivors had significantly (one-way ANOVA) increased respiratory rate, percent neutrophils on complete blood count, hemoglobin, INR, C-reactive protein, and bilirubin and significantly decreased temperature, systolic blood pressure, platelets, sodium, albumin, calcium (corrected for albumin), base excess and bicarbonate (all *P *= 0.05).

## Conclusion

The implications of the apparent differences noted between nonsurvivors and survivors in this survey of cases from the first known outbreak of injectional anthrax require further study. However, these differences might inform the design of research during future outbreaks or of methods to identify patients most in need of anthrax-specific therapies such as toxin-directed antibodies.

